# Effects of sleep pattern, duration, and quality on premenstrual syndrome and primary dysmenorrhea in korean high school girls

**DOI:** 10.1186/s12905-023-02600-z

**Published:** 2023-08-28

**Authors:** Daye Jeong, Heakyong Lee, Jaehee Kim

**Affiliations:** Graduate School of Alternative Medicine, Kyonggi University (Seoul Campus), 24, Kyonggidae- ro 9-gil, Seodaemun-gu, Seoul, 03746 Republic of Korea

**Keywords:** Dysmenorrhea, Premenstrual syndrome, Sleep, Adolescents, COVID-19

## Abstract

**Background:**

Sleep deprivation is known to be a risk factor for premenstrual syndrome and primary dysmenorrhea in adults. However, it has rarely been investigated in adolescents. The aim of this study was to investigate whether sleep pattern, duration, and quality independently affect premenstrual syndrome and primary dysmenorrhea in adolescent girls. An additional purpose was to investigate the sleep status in Korean adolescent girls during the COVID-19 pandemic.

**Methods:**

A cross-sectional survey study was conducted in 519 high school girls aged 15 to 18 years in Gyeonggido, South Korea, in 2021 during the COVID-19 lockdown. Menstrual pain intensity and menstrual symptoms were assessed using the visual analogue scale (VAS) and Cox menstrual symptom scale (CMSS), respectively. Premenstrual syndrome was assessed by the premenstrual symptoms screening tool (PSST). Sleep was assessed by the Pittsburgh Sleep Quality Index (PSQI). The known risk factors of dysmenorrhea, including menstrual and lifestyle characteristics and stress, were assessed as covariates.

**Results:**

During the pandemic, approximately 68% of girls slept 7 h or less, while about 60% reported poor sleep quality. Additionally, 64% of participants had a bedtime later after 1AM, and 34% woke up later after 8AM. Late bedtime significantly affected VAS (P = 0.05), CMSS severity and frequency (both P < 0.01), and PSST symptom (P < 0.01). Waking up late affected CMSS severity (P < 0.05), PSST symptom (P = 0.05), and PSST function (P < 0.05). However, the significance of these effects disappeared after controlling for covariates. Sleeping less than 5 h affected CMSS frequency (P < 0.05) and PSST symptoms (P < 0.001). After controlling for covariates, the significance of the effect on PSST symptom remained (P < 0.05). General sleep quality and PSQI components, including subjective sleep quality, sleep latency, sleep disturbance, use of sleeping medication, and daytime dysfunction, significantly affected CMSS frequency and severity and PSST symptom after controlling for covariates (P < 0.05, P < 0.01, or P < 0.001). The multiple regression analysis revealed that among sleep characteristics, sleep quality was the most important risk factor of premenstrual syndrome and dysmenorrhea.

**Conclusion:**

Our study result heightens the importance of healthy sleep hygiene, especially sleep quality in the management of premenstrual syndrome and dysmenorrhea in adolescent girls.

## Introduction

The prominent menstrual problems are dysmenorrhea, which refers to menstrual pain occurring during menstruation, and premenstrual syndrome (PMS), which starts during the luteal phase of menses and disappears with the onset of menstruation [1, 2]. The prevalence of dysmenorrhea and PMS varies across countries and study reports, but two meta-analysis studies reported that the global prevalence of dysmenorrhea and moderate-to-severe PMS was 71.1% and 47.8%, respectively [1, 3]. Dysmenorrhea is subcategorized into primary, without pelvic pathology, and secondary, caused by another disease, and primary dysmenorrhea is more common [4]. The etiology of primary dysmenorrhea and PMS is multifactorial and not fully understood [4, 5]. Accordingly, multidisciplinary approaches have been taken to alleviate menstrual pain and PMS [5, 6]. These approaches include evaluating modifiable risk factors and maintaining these factors positively [6, 7].

Sleep habits have been considered as one of the main modifiable lifestyle risk factors for dysmenorrhea and PMS [7, 8] although it is not clear whether the menstrual problems cause poor sleep or vice versa [8, 9]. Given that, sleep and menstrual problems could affect each other and form a vicious circle [8, 10]. Nonetheless, recent reports indicate that sleep characteristics such as poor sleep quality, insomnia, and short sleep duration, are related to dysmenorrhea and PMS in adults [8, 10–13]. Sleep disruption could worsen dysmenorrhea by promoting inflammation, which is one of the underlying causes of dysmenorrhea [14, 15]. Considering that poor sleep can exacerbate stress, a known risk factor for dysmenorrhea and PMS [8, 16, 17], it is possible that stress partly mediates the impact of sleep on them. Furthermore, melatonin, a hormone associated with reproductive health [18, 19], may also play a role: sleep disturbances have been reported to reduce melatonin production, and decreased melatonin levels have been linked to menstrual problems [20, 21]. Taken together, these findings suggest that sleep could affect dysmenorrhea and PMS, but its impact on dysmenorrhea and PMS in adolescents has been rarely investigated.

Since compared to adults, adolescents have different appropriate sleep time and sleep pattern including longer sleep time, later bedtime, and later wake-up time [22, 23], it is necessary to examine whether sleep characteristics affect dysmenorrhea and PMS in adolescents. In general, a shift in sleep/wake patterns occurs in late adolescents [24]. High school students have an earlier wake time due to school schedules and a later bedtime due to diverse reasons, including academic demands, entertainments, and employment, resulting in the decreased sleep time [9, 24]. Particularly, Korean high school students experience severe sleep deprivation with an average sleep time of 5–6 h, because of academic demands and early school start time, along with more daytime sleepiness and more sleep/wake-problem behavior compared to early adolescents [25].

These severe sleep deprivation and irregular sleep/wake patterns may worsen dysmenorrhea and PMS. Additionally, sleep quality is known to be a risk factor for dysmenorrhea and PMS in adults [10, 11, 13]. However, the effect of sleep quality and sleep pattern on menstrual problems has not been investigated in adolescents. Studies on adolescents have reported that shorter sleep duration is a risk factor for the menstrual pain [26, 27]. Adolescent girls with dysmenorrhea also reported higher scores of insomnia, daytime sleepiness, and sleep apnea [28]. Yoshimi et al. [29] also reported that sleep latency is a risk factor for PMS of high school students.

Moreover, the measurement methods of dysmenorrhea and PMS used in the previous studies regarding sleep and menstrual problems were unidimensional (i.e., mostly “do you have dysmenorrhea?”). Therefore, there may be a lack of information on the effect of sleep on the multidimensional aspects of these menstrual problems. Since dysmenorrhea is a multidimensional phenomenon, the use of a multidimensional scoring scale has been recommended, especially in adolescents [30]. Adolescents may be less skilled at recalling the accurate time and intensity of menstrual symptoms because they are still learning about their menstrual cycle [31]. Accordingly, the use of multidimensional measurements to assess dysmenorrhea and PMS seems to be more appropriate in the adolescent population.

The lockdown and social distancing resulted from the outbreak of the Coronavirus Disease 2019 (COVID-19) brought various changes to adolescents’ daily life [32, 33]. This included increased smartphone use, online classes, and restriction of outdoor activities, which could affect sleep [34, 35]. Indeed, deterioration of sleep was observed in adolescents during the lockdown period related to the COVID-19 pandemic in Austria and England [32, 36]. Lately, it has been reported that the COVID-19 pandemic aggravated menstrual problems showing a significant increase in PMS, heavy menstrual bleeding, painful periods, and missed periods compared to before pandemic in adult women [37]. Maher et al. [38] reported that increased poor sleep caused overall changes in menstrual cycle and missed periods during the pandemic in adults. However, the association between sleep characteristics and menstrual problems during the COVID-19 pandemic has not been investigated in adolescents.

Some factors have been reported to be associated with a higher risk of dysmenorrhea, including age, obesity, family history of dysmenorrhea, an earlier age at menarche, long menstrual periods, heavy menstrual flow, irregular and longer menstrual cycles, smoking, alcohol consumption, caffeine consumption, skipping breakfast, and heavy stress [4, 8, 27, 31].

Therefore, the primary purpose of this study was to investigate whether sleep pattern, duration, and quality independently affect PMS and dysmenorrhea, while controlling for other dysmenorrhea-related risk factors in high school girls during the COVID-19 pandemic. The secondary purpose of the study was to investigate changes in sleep characteristics during the COVID-19 pandemic in Korean high school girls. We examined the diverse aspects of sleep and identified which aspect independently affected adolescents’ PMS and dysmenorrhea, using validated standardized measurements of sleep and menstrual variables.

## Methods

### Study design and participants

**Fig. 1:**
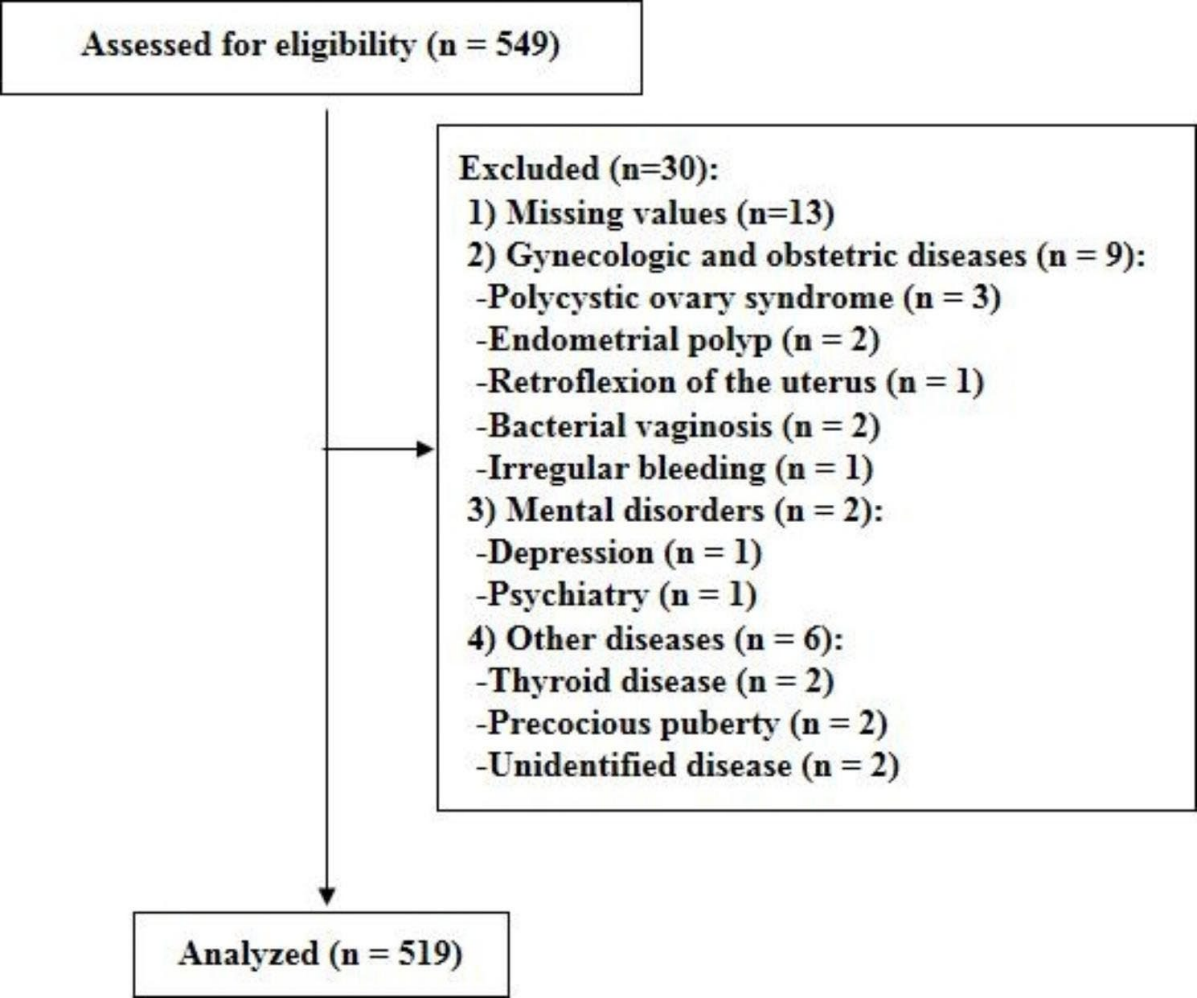
The study flowchart

We performed a cross-sectional study. Survey was conducted at high schools located in Goyang City and Paju City of Gyeonggido in South Korea in August and September 2021, during the COVID-19 lockdown. The flowchart of the study is presented in Fig. 1. A total of 549 adolescent girls in the 1st to 3rd year of high school completed the survey. Data of 30 participants were excluded from the analysis due to the following reasons: missing data for variables of menstrual characteristics, dysmenorrhea, or psychological factors (n = 13). Seventeen subjects were further excluded because they have gynecologic and obstetric diseases or mental disorders which could potentially affect the outcome: polycystic ovary syndrome (n = 3), endometrial polyp (n = 2), retroflexion of the uterus (n = 1), bacterial vaginosis (n = 2), irregular bleeding (n = 1), depression (n = 1), psychiatry (n = 1), thyroid disease (n = 2), precocious puberty (n = 2), and unidentified disease (n = 2).

We designed two independent studies including the present study using the same population. The ethical approval for the study was obtained from Institutional Review Board (IRB) of Kyonggi University (KGU-20,210,222-HR-065-03). Informed consent was obtained from all subjects. The paper regarding effect of positive and negative psychological factors on dysmenorrhea is in press [39]. This study was exempt from IRB of Kyonggi University (KGU-20,230,104-HR-097). The data of sleep and PMS were not previously published.

### Risk factors of dysmenorrhea

The known risk factors for the dysmenorrhea included age, body mass index, menstrual and lifestyle characteristics, and stress level [8, 31]: early menarche before 12 years, family history of dysmenorrhea (no history or unknown vs. having history); abnormal length of menstrual cycle (≤ 35days vs. >35days); regularity of menstrual cycle (regular vs. irregular), heavy volume of menstrual fluid (light-to-moderate vs. heavy); long period length (≤ 7days vs. >7days); skipping breakfast (not eating breakfast vs. eating breakfast once or more); drinking caffeine beverage (none vs. drinking once or more); smoking (current or past smoker vs. non-smoker); drinking (current drinker vs. non-drinker). Perceived stress level was evaluated using the Korean version of 10-item perceived stress scale (PSS) [40]. A higher score of total PSS score (range 0–40) indicates a greater stress level [41].

### Dysmenorrhea and premenstrual syndrome

Dysmenorrhea was evaluated by measuring pain intensity and menstrual symptoms experienced during the period [30]. Menstrual pain intensity was assessed using a visual analog scale (VAS). The VAS consists of a 10-cm horizontal line and the ends of the line are labeled as “no pain” and “most severe pain” [30]. Participant was instructed to mark her pain intensity on the line. The distance from the left end was measured in centimeters.

Menstrual symptom related to dysmenorrhea was evaluated using the Korean version of Cox menstrual symptom scale (CMSS) [42, 43]. The CMSS evaluates the severity and frequency of 18 dysmenorrhea symptoms including cramps, nausea, vomiting, loss of appetite, headaches, backaches, leg aches, dizziness, weakness, diarrhea, facial blemishes, abdominal pain, flushing, sleeplessness, general aching, depression, irritability, and nervousness [42, 43]. The severity and frequency of symptoms were scored and added up, separately. The severity of each symptom is scored on a 5-point scale (0–4): not noticeable (0); slightly bothersome (1); moderate bothersome (2); severely bothersome (3); very severely bothersome (4) [42]. The scale of frequency ranges 0–4: the symptom did not occur (0); lasted less than 3 h (1); lasted 3–7 h (2); lasted an entire day (3); lasted several days (4) [42].

The PMS was evaluated using the Korean version of premenstrual symptoms screening tool (PSST) [44, 45]. The PSST consists of 14 physical, psychological, and behavioral symptoms and 5 measures on how these symptoms interfered with her ability to function in daily activities (functional impairments) due to the PMS [44]. The PSST symptoms listed are as follow: anger/irritability; anxiety/tension; tearful/increased sensitivity to rejection; depressed mood/hopelessness; decreased interest in school activities; decreased interest in home activities; decreased interest in social activities; difficulty concentrating; fatigue/lack of energy; overeating/food cravings; insomnia; hypersomnia; feeling over-whelmed or out of control; physical symptoms (breast tenderness, headaches, joint/muscle pain, bloating, weight gain) [46]. Functional impairments include school/work efficiency or productivity, relationships with friends, classmates/coworkers, relationship with family, social life activities, and home responsibilities [46]. The scale of each item ranges 0–3: not at all (0); mild (1); moderate (2); severe (3) [44]. The number of 14 symptoms and 5 functional impairments, which was reported as moderate to severe, was counted separately [44]. The Korean PSST had good reliability [45].

### Sleep pattern, duration, and quality

Sleep variables were assessed using the Korean version of the Pittsburgh Sleep Quality Index (PSQI), which was proven to be valid and reliable [47, 48]. The PSQI evaluates sleep quality and disturbances over a one-month period and consists of seven components: subjective sleep quality, sleep latency, sleep duration, habitual sleep efficiency, sleep disturbances, use of sleeping medication, and daytime dysfunction [47]. The score of each component ranges 0–3 and the global score is the sum of scores for these seven components with a range of 0–21 [49]. The global score of more than 5 indicates poor sleep quality [49]. Developers have suggested that a global PSQI score > 5 yields a diagnostic sensitivity of 89.6% and a specificity of 86.5% in distinguishing patients with sleep problems from healthy controls in their validation study [49].

Additionally, sleep duration, bedtime, and wake-up time were measured using the following PSQI items: “During the past month, how many hours of actual sleep did you get at night?”; “During the past month, when what have you usually gone to bed at night?”; “During the past month, when have you usually gotten up in the morning?” [49].

Sleep duration was categorized as less than 5 h or 5 h or more for regression analyses to identify risk factors based on the following reasons. The PSQI component of sleep duration is scored as follows: more than 7 h of sleep receives a score of 0 (indicating the best), whereas less than 5 h of sleep is assigned a score of 3 (indicating the worst) [47, 49]. It has been reported that Korean high school students sleep for around 5 h, which is less than the recommended 8 h of sleep for adolescents [25]. Furthermore, the independent variable of sleep duration in hours was not significant in the regression analyses.

It has been reported that Korean high school students exhibit irregular sleep patterns, with an average bedtime of 1 AM [25]. Bedtime and wake-up time were categorized based on sleep characteristics of the present study’s data, which were collected during the COVID-19 lockdown. Only 22 out of 519 adolescents slept before 12 PM in the present study. Bedtime was categorized as 10 PM to 1 AM or after 1 AM (approximately two-thirds of participants), and wake-up time was categorized as 5 AM to 8 AM (approximately two-thirds of participants) or after 8 AM.

### Statistical analysis

The data was analyzed using SPSS version 27.0 (IBM SPSS Statistics, Armonk, NY, US). The significance level was set at 0.05 for all analyses. A p-value of 0.05 or lower was considered as statistically significant. The independent t-test and Chi-square test were used to test the significance of group-difference in participants’ characteristics according to sleep quality. Significance of group-difference in menstrual variables according to sleep characteristics was tested using the independent t-test and one-way ANOVA with Scheffe’s multiple comparison tests. Correlations between sleep variables and menstrual variables were assessed using Pearson correlation coefficients (r).

Simple and multiple linear regression analyses were used to test the effects of sleep variables on menstrual variables. A separate simple linear regression analysis was conducted with bedtime (10PM-1AM vs. after 1AM), wake-up time (5AM-8AM vs. after 8AM), sleep duration (< 5 h vs. ≥5 h), sleep quality (good vs. poor), and 7 PSQI component scores (range 0 to 3) as an independent variable to identify sleep factor that influences dysmenorrhea and PMS. Dependent variables included VAS, CMSS severity, CMSS frequency, number of moderate-to-severe PSST symptoms, and number of moderate-to-severe PSST functional impairments. Additionally, multiple linear regression analyses were performed to identify if the effect of each sleep factor was significant after controlling for other risk factors for dysmenorrhea (covariates), including family history of dysmenorrhea, having menarche before 12 years, regularity of menstrual cycle, length of menstrual cycle, menstrual volume, period length, stress, caffeine consumption, eating breakfast, smoking, and drinking. In the multiple regression analyses, the presence of multicollinearity among the independent variables was evaluated with tolerance with cut-off points of more than 0.1 and the variation inflation factor (VIF < 10). Multicollinearity problems were not found among the independent variables in all multiple regression analyses.

Finally, multiple regression analyses were performed to identify the most important sleep-related risk factors of dysmenorrhea and PMS. Bedtime, wake-up time, sleep duration, and sleep quality were entered together with all covariates in the multiple regression model.

## Results

### Participant characteristics

Most participants had dysmenorrhea, with 17.3% (n = 90) experiencing mild pain (0 < VAS < 3) and 76.5% (n = 397) experiencing moderate-to-severe pain (3 ≦ VAS), while 6.2% had no pain (VAS = 0) when dysmenorrhea was defined by the intensity of menstrual pain measured by VAS [36]. Regarding classification of PMS defined by PSST [40, 50], 60.9% of participants (n = 316) reported either no symptoms or mild symptoms, whereas 39.1% of them (n = 203) experienced moderate-to-severe PMS symptoms. Additionally, 4.8% of the participants (n = 25) did not report any of the 14 PMS symptoms and 5 functional impairments.

Characteristics of 519 study participants are presented in Table 1. The study participants were high-school students (1st year to 3rd year) with a mean age of 16.2 ± 0.8 years (aged 15 to 18 years). Most participants had their menarche after 12 years (71.7%) and had a normal length of menstrual cycle (89.2%) and period length (86.7%). About half of them had an irregular menstrual cycle, and 22.5% of participants had heavy menstrual volume. About 36% of them had a family history of dysmenorrhea. The majority of participants were non-smokers (94.4%), did not drink alcohol beverages (79.8%), ate breakfast (77.8%), consumed caffeine beverages (60.9%), and did not engage in moderate or vigorous exercise (79.4%). The average PSS score of participants was 20.6 ± 6.1, and the mean sleep duration of participants was 6.2 ± 1.6 h. Overall, 59.7% of the total sample had a global PSQI score of more than 5, indicating poor sleep quality.

Differences in participant characteristics between the groups with good and poor sleep quality are presented in Table 1. Among menstrual and lifestyle characteristics, period regularity (P < 0.001), volume of menstrual fluid (P < 0.01) and caffeine consumption (P < 0.01) showed the significant group-differences, revealing that proportions of irregular menstrual cycle, heavy menstrual volume, and caffeine consumer were higher in the group with poor sleep quality. There were no group-differences in all other menstrual and lifestyle characteristics, age, and BMI. Significant group-differences were observed in PSS, sleep duration, and the global PSQI score, as well as average scores for the 7 PSQI components (P < 0.01 or P < 0.001), revealing a higher stress level, higher PSQI scores, and shorter sleep time in the group with poor sleep quality.

**Table 1:** Participant characteristics

Variables	Categories	Total (N = 519*)*	Sleep quality		*P* ^b^
			Good (N = 209)^§^	Poor (N = 310)	
Age (years)		16.20 ± 0.81	16.21 ± 0.57	16.20 ± 0.46	0.937
Body mass index (kg/m^2^) ^a,^		20.06 ± 2.88	20.06 ± 0.21	20.06 ± 0.18	0.981
Menarcheal age	≥ 12 years	372(71.7%)	151(72.2%)	221(71.3%)	0.812
	< 12 years	147(28.3%)	58(27.8%)	89(28.7%)	
Period regularity	Regular	282(54.3%)	137(65.6%)	145(46.8%)	0.000^***^
	Irregular	237(45.7%)	72(34.4%)	165(53.2%)	
Length of menstrual cycle	≤ 35 days	463(89.2%)	183(87.6%)	280(90.3%)	0.320
	> 35 days	56(10.8%)	26(12.4%)	30(9.7%)	
Volume of menstrual fluid	Light/moderate	402(77.5%)	177(84.7%)	225(72.6%)	0.001^**^
	Heavy	117(22.5%)	32(15.3%)	85(27.4%)	
Period length	≤ 7 days	450(86.7%)	177(84.7%)	273(88.1%)	0.267
	> 7 days	69(13.3%)	32(15.3%)	37(11.9%)	
Family history of dysmenorrhea	No/ Unknown	335(64.5%)	130(62.2%)	205(66.1%)	0.359
	Yes	184(35.5%)	79(37.8%)	105(33.9%)	
Smoking	No	490(94.4%)	199(95.2%)	291(93.9%)	0.513
	Yes	29(5.6%)	10(4.8%)	19(6.1%)	
Drinking	No	411(79.8%)	170(81.3%)	244(78.7%)	0.464
	Yes	105(20.2%)	39(18.7%)	66(21.3%)	
Eating breakfast	No	115(22.2%)	41(19.6%)	74(23.9%)	0.252
	Yes	404(77.8%)	168(80.4%)	236(76.1%)	
Caffeine consumption	No	203(39.1%)	96(45.9%)	107(34.5%)	0.009^**^
	Yes	316(60.9%)	113(54.1%)	203(65.5%)	
Moderate-to-vigorous exercise	No	412(79.4%)	170(81.3%)	242(78.1%)	0.366
	Yes	107(20.6%)	39(18.7%)	68(21.9%)	
Perceived stress		20.58 ± 6.07	18.22 ± 0.41	22.16 ± 0.32	0.000^***^
Sleep duration (hours)		6.20 ± 1.57	6.85 ± 0.98	5.76 ± 0.09	0.000^***^
Pittsburgh sleep quality index					
Global score		6.35 ± 2.71	3.86 ± 0.08	8.04 ± 0.12	0.000^***^
Subjective sleep quality		1.13 ± 0.66	0.74 ± 0.33	1.40 ± 0.36	0.000^***^
Sleep latency		1.09 ± 0.99	0.49 ± 0.05	1.50 ± 0.05	0.000^***^
Sleep duration		1.17 ± 1.04	0.68 ± 0.05	1.51 ± 0.06	0.000^***^
Habitual sleep efficiency		0.21 ± 0.58	0.03 ± 0.01	0.33 ± 0.04	0.000^***^
Sleep disturbance		0.87 ± 0.52	0.67 ± 0.03	1.01 ± 0.03	0.000^***^
Use of sleeping medication		0.06 ± 0.32	0.02 ± 0.01	0.09 ± 0.02	0.007^**^
Daytime dysfunction		1.08 ± 0.87	1.23 ± 0.06	2.19 ± 0.04	0.000^***^

Data are presented as N (%) or mean ± standard deviation. ^a^N = 455. ^b^Significance of the group difference was tested using the independent t-test or the Chi-square test. ^**^P < 0.01, ^***^P < 0.001.

### Sleep and menstrual characteristics during the COVID-19 pandemic

The sleep pattern of Korean high school girls during the COVID-19 pandemic and menstrual differences according to sleep pattern are shown in Table 2. The proportion of participants who went to bed between 10PM and 1AM was 36%, and the rest of them slept after 1AM. The CMSS severity and frequency, as well as the number of moderate-to-severe PSST symptoms, were higher in girls who slept after 1AM, compared to those who slept earlier (all P < 0.01). Most girls got up between 5AM and 8AM (66.5%),while 33.5% of them woke up later.

Girls who got up late had worse CMSS severity (P < 0.05) and PSST functional impairments (P < 0.05). Approximately 60% of high school girls had a poor sleep quality. The VAS, CMSS severity and frequency, and PSST symptoms and functional impairment were worse in girls with poor sleep quality compared to those with good sleep quality (P < 0.01 or P < 0.001). Half of the girls (53.6%) slept 5–7 h, 32.4% of them slept more than 7 h, and 14.1% slept less than 5 h. The CMSS frequency tended to be higher in girls who slept less than 5 h compared to those who slept more than 7 h (P = 0.06). Additionally, the number of moderate-to-severe PSST symptoms was higher in girls who slept less than 5 h, compared to those who slept 5–7 h or more than 7 h (both P < 0.01).

### Correlations between sleep and menstrual variables

The results of Pearson correlation coefficients are presented in Table 3. Sleep duration was not associated with any of the five menstrual variables related to dysmenorrhea and premenstrual symptoms. The correlations between the global PSQI score and all menstrual variables (VAS, CMSS severity and frequency, and number of PSST symptoms and functional impairment) were found to be significant (all P < 0.001), indicating that poorer sleep quality was associated with more severe dysmenorrhea and premenstrual symptoms. All five menstrual variables were positively correlated with five of the seven PSQI component scores: subjective sleep quality, sleep latency, sleep disturbance, use of sleeping medication, and daytime dysfunction (P < 0.05, P < 0.01 or P < 0.001), showing that better scores on these components were associated with less severe dysmenorrhea and premenstrual symptoms. The PSQI sleep duration was associated with the number of moderate-to-severe PSST symptoms (P < 0.05).

### Impacts of sleep variables on menstrual variables

The result of significant unstandardized and standardized regression coefficients from simple and multiple regression analyses are presented in Table 4 (Vertical line of the first row should be adjusted as shown in Table 5.). The results of simple regression analyses regarding effect of bedtime (10PM-1AM vs. After 1AM) on variables of dysmenorrhea and premenstrual symptoms showed that bedtime affected VAS (P = 0.05), the CMSS severity and frequency (both P < 0.01), and the number of PSST symptoms (P < 0.01). These findings indicate that going to bed late after 1AM increased menstrual pain intensity, the severity and frequency of menstrual symptoms, and the number of moderate-to-severe premenstrual symptoms. However, the results of multiple linear regression analyses showed that the significance of the effect of bedtime disappeared after controlling for other risk factors for dysmenorrhea (covariates).

The unadjusted β of wake-up time was significant for CMSS severity (P < 0.05) and the number of PSST symptoms (P = 0.05) and functional impairment (P < 0.05), indicating that getting up between 5AM and 8AM was associated with less severe menstrual symptoms and a lower occurrence of moderate-to-severe premenstrual symptoms and functional impairment, compared to getting up later in the morning and in the afternoon. However, when covariates were entered, the adjusted β was not significant.

**Table 2:** Sleep and menstrual characteristics

Sleep Variables	Categories	N (%)	VAS	CMSS severity	CMSS frequency	No. of PSST symptom	No. of PSST functional impairment
Bedtime	10PM-1AM	187 (36.0%)	5.24 ± 0.21	14.51 ± 0.77	16.62 ± 0.89	4.06 ± 0.28	1.03 ± 0.11
	After 1AM	332 (64.0%)	5.75 ± 0.15	17.58 ± 0.61	19.83 ± 0.72	5.18 ± 0.22	1.20 ± 0.08
	*P* ^a^		0.050	0.002^**^	0.006^**^	0.002^**^	0.197
Wake-up time	5AM-8AM	345 (66.5%)	5.48 ± 0.15	15.74 ± 0.59	18.09 ± 0.70	4.53 ± 0.22	1.04 ± 0.74
	After 8AM	174 (33.5%)	5.75 ± 0.23	17.95 ± 0.83	19.82 ± 0.95	5.26 ± 0.30	1.34 ± 0.12
	*P* ^a^		0.313	0.031^*^	0.147	0.052	0.039^*^
Sleep quality	Good	209 (40.3%)	4.86 ± 0.20	12.63 ± 0.65	14.11 ± 0.71	3.33 ± 0.24	0.78 ± 0.82
	Poor	310 (59.7%)	6.05 ± 0.15	19.07 ± 0.64	21.75 ± 0.76	5.75 ± 0.23	1.39 ± 0.09
	*P* ^a^		0.001^**^	0.000^***^	0.000^***^	0.000^***^	0.000^***^
Sleep duration	> 7 h	168 (32.4%)	5.48 ± 0.23	16.51 ± 0.88	17.76 ± 0.94	4.45 ± 0.31	1.17 ± 0.12
	5–7 h	278 (53.6%)	5.54 ± 0.17	15.90 ± 0.62	18.35 ± 0.74	4.55 ± 0.24	1.07 ± 0.09
	< 5 h	73 (14.1%)	5.90 ± 0.35	18.63 ± 1.39	22.03 ± 1.73	6.40 ± 0.48c,d	1.34 ± 0.16
	*P* ^b^		0.549	0.166	0.049^*^	0.001^**^	0.355

VAS: visual analogue scale, CMSS: Cox menstrual symptom scale, No.: number, PSST: premenstrual symptoms screening tool. ^a^Significance of the group difference was tested using the independent t-test. ^b^Significance of the group difference was tested using one-way ANOVA. ^c^Significantly different from ‘> 7 hours’ by Scheffe’s multiple comparison tests., ^d^Significantly different from ‘5–7 hours’ by Scheffe’s multiple comparison tests. ^*^P < 0.05, ^**^P < 0.01, ^***^P < 0.001

**Table 3:** Pearson correlation coefficient (r) values for the relationships between sleep and menstrual variables

Sleep variables	VAS	CMSS severity	CMSS frequency	No. of PSST symptom	No. of PSST functional impairment
Sleep duration (hours)	-0.011	0.025	-0.016	-0.067	0.019
Global PSQI score	0.225^***^	0.337^***^	0.353^***^	0.374^***^	0.288^***^
Subjective sleep quality	0.194^***^	0.296^***^	0.268^***^	0.283^***^	0.234^***^
Sleep latency	0.144^***^	0.194^***^	0.195^***^	0.214^***^	0.184^***^
PSQI sleep duration	0.012	0.006	0.06	0.109^*^	0.006
Habitual sleep efficiency	0.041	0.029	0.041	0.083	0.044
Sleep disturbance	0.235^***^	0.359^***^	0.342^***^	0.290^***^	0.286^***^
Use of sleeping medication	0.107^*^	0.162^***^	0.171^***^	0.137^**^	0.136^**^
Daytime dysfunction	0.169^***^	0.304^***^	0.307^***^	0.299^***^	0.253^***^

VAS: visual analogue scale, CMSS: Cox menstrual symptom scale, No.: number, PSST: premenstrual symptoms screening tool, PSQI: Pittsburgh sleep quality index. N = 519. ^*^P < 0.05, ^**^P < 0.01, ^***^P < 0.001

**Table 4:** Effect of sleep pattern and quality on dysmenorrhea and premenstrual syndrome

Dependent variables		VAS		CMSS severity			CMSS frequency			No. of PSST symptom			No. of PSST functional impairment	
Independent variables		Regression coefficients in the simple regression model												
		B (SE)	β		B (SE)	β		B (SE)	β		B (SE)	β	B (SE)	β
Bedtime		0.510(0.260)	**0.086** ^**b**^		3.064 (0.996)	**0.134** ^**^		3.208(1.165)	**0.120** ^**^		1.119(0.366)	**0.133** ^**^	0.173(0.134)	0.057
Wake-up time		0.267(0.265)	0.044		2.206(1.017)	**0.095** ^*^		1.729(1.191)	0.064		0.728(0.374)	**0.085** ^**b**^	0.296(0.136)	**0.095** ^*^
Sleep duration		0.357(0.360)	0.047		2.503(1.383)	0.079		3.904(1.611)	**0.106** ^*^		1.888(0.502)	**0.163** ^***^	0.233 (0.185)	0.055
Sleep quality		1.194(0.250)	**0.206** ^***^		6.437(0.942)	**0.288** ^***^		7.646(1.098)	**0.293** ^***^		2.418(0.345)	**0.295** ^***^	0.615(0.128)	**0.206** ^***^
PSQI Components	Subjective sleep quality	0.840(0.187)	**0.194** ^***^		4.927(0.701)	**0.296** ^***^		5.214(0.825)	**0.268** ^***^		1.731(0.258)	**0.283** ^***^	0.521(0.095)	**0.234** ^***^
	Sleep latency	0.413(0.125)	**0.144** ^**^		2.142(0.478)	**0.194** ^***^		2.519(0.558)	**0.195** ^***^		0.871(0.175)	**0.214** ^***^	0.271(0.064)	**0.184** ^***^
	Sleep disturbance	1.293(0.235)	**0.235** ^***^		7.609(0.870)	**0.359** ^***^		8.481(1.023)	**0.342** ^***^		2.257(0.328)	**0.290** ^***^	0.809(0.119)	**0.286** ^***^
	Daytime dysfunction	0.551(0.141)	**0.169** ^***^		3.811(0.525)	**0.304** ^***^		4.492(0.613)	**0.307** ^***^		1.375(0.193)	**0.299** ^***^	0.424(0.071)	**0.253** ^***^
	Use of sleep medication	0.932(0.382)	**0.107** ^*^		5.443(1.460)	**0.162** ^***^		6.730(1.703)	**0.171** ^***^		1.686(0.538)	**0.137** ^**^	0.609(0.196)	**0.136** ^**^
		**Regression coefficients adjusted for covariates in the multiple regression model** ^a^												
**Sleep variables**		**B (SE)**	**β**		**B (SE)**	**β**		**B (SE)**	**β**		**B (SE)**	**β**	**B (SE)**	**β**
Bedtime		0.179(0.247)	0.03		1.271(0.877)	0.056		1.179(1.052)	0.044		0.469(0.326)	0.056	0.012(0.126)	-0.004
Wake-up time		0.020(0.251)	0.003		0.770(0.889)	0.033		0.336(1.067)	0.012		0.195(0.331)	0.023	0.134(0.127)	0.043
Sleep duration		0.034(0.345)	-0.004		0.007(1.225)	0		1.092(1.468)	0.03		0.976(0.453)	**0.084** ^*^	0.019(0.175)	-0.005
Sleep quality		0.481(0.258)	0.083		3.099(0.908)	**0.139** ^**^		4.168(1.085)	**0.160** ^***^		1.155(0.337)	**0.141** ^**^	0.232(0.131)	0.078
PSQI components	Subjective sleep quality	0.298(0.192)	0.069		2.242(0.676)	**0.134** ^**^		2.337(0.813	**0.120** ^**^		0.726(0.252	**0.119** ^**^	0.227(0.097)	**0.102** ^*^
	Sleep latency	0.210(0.122)	0.073		1.084(0.432)	**0.098** ^*^		1.455(0.518)	**0.113** ^**^		0.540(0.160)	**0.133** ^**^	0.164(0.062)	**0.111** ^**^
	Sleep disturbance	0.749(0.235)	**0.136** ^**^		4.848(0.817)	**0.229** ^***^		5.467(0.983)	**0.221** ^***^		1.211(0.309)	**0.156** ^***^	0.516(0.119)	**0.183** ^***^
	Daytime dysfunction	0.176(0.143)	0.054		2.013(0.502)	**0.161** ^***^		2.574(0.601)	**0.176** ^***^		0.616(0.188)	**0.134** ^**^	0.222(0.072)	**0.133** ^**^
	Use of sleep medication	0.496(0.361)	0.057		3.494(1.276)	**0.104** ^**^		4.607(1.528)	**0.117** ^**^		0.976(0.476)	**0.079** ^*^	0.392(0.183)	**0.087** ^*^

N = 519. VAS: visual analogue scale, CMSS: Cox menstrual symptom scale, No.: number, PSST: premenstrual symptoms screening tool. Bedtime: 10PM-1AM *vs*. after 1AM; Wake-up time: 5AM-8AM *vs*. after 8AM; Sleep duration: < 5 hours *vs*. ≥5 hours; Sleep quality: Good *vs*. poor; The scale of each PSQI component range 0 to 3. ^a^The entered covariates for multiple regression analyses were as follow: family history of dysmenorrhea, early menarche before 12 years, regularity of menstrual cycle, length of menstrual cycle, menstrual volume, period length, stress, caffeine consumption, eating breakfast, smoking, and drinking. ^*^P < 0.05, ^**^P < 0.01, ^***^P < 0.001. ^b^P = 0.05. The significant values are in bold

The unadjusted β of sleep duration (< 5 h vs. ≥5 h) for CMSS frequency (P < 0.05) and PSST symptoms (P < 0.001) were significant, indicating that sleeping less than 5 h was associated with more frequent occurrence of menstrual symptoms and worse premenstrual symptoms. After controlling for covariates, only the adjusted β for PSST symptoms remained significant (P < 0.05).

The results of simple regression analyses showed that general PSQI sleep quality (poor vs. good) had significant effects on all 5 dependent variables, including VAS, CMSS severity and frequency, and the number of PSST symptoms and functional impairment (all P < 0.001). These results indicated that girls with poor sleep quality experienced more severe menstrual pain and symptoms, more frequent menstrual symptoms, and more severe premenstrual symptoms and functional impairments. In multiple regression analyses, after controlling for covariates, the effect of sleep quality remained significant for the CMSS severity (P < 0.01) and frequency (P < 0.001), as well as the number of moderate-to-severe PSST symptoms (P < 0.01).

Among the 7 PSQI components, 5 components (subjective sleep quality, sleep latency, sleep disturbance, daytime dysfunction, and use of sleep medication) significantly affected all 5 dependent variables (P < 0.05, P < 0.01, or P < 0.001), showing that higher components scores resulted in worse dysmenorrhea and premenstrual symptoms. After controlling for covariates, all 5 PSQI components significantly affected the CMSS frequency and severity and PSST symptoms and functional impairments (P < 0.05, P < 0.01, or P < 0.001), while only sleep disturbance significantly affected VAS (P < 0.01). The PSQI sleep duration and habitual sleep efficiency were not significant factors affecting dysmenorrhea and premenstrual symptoms.

### The most important sleep-related risk factors of dysmenorrhea and premenstrual symptoms

To examine which variables were the most important risk factors of dysmenorrhea and premenstrual symptom among sleep variables, multiple regression analyses were performed. Bedtime, wake-up time, sleep duration, and general sleep quality were entered together in the regression model with all covariates. As shown in Table 5, the results of multiple regression analyses showed that the significant risk factors of VAS included PSS (P < 0.001) and menstrual volume (P < 0.001). The significance of the adjusted beta coefficient for sleep quality was close to the significance level (P = 0.058). The significant risk factors of the CMSS severity included general sleep quality (P < 0.001), PSS (P < 0.001), menstrual volume (P < 0.001), family history of dysmenorrhea (P < 0.001), having menarche before 12 years (P < 0.05), eating breakfast (P < 0.01), and drinking (P < 0.01). The significant risk factors of the CMSS frequency included general sleep quality (P < 0.001), PSS (P < 0.001), menstrual volume (P < 0.001), family history of dysmenorrhea (P < 0.01), and eating breakfast (P < 0.01).

The significant risk factors of the PSST symptoms included general sleep quality (P < 0.01), PSS (P < 0.001), menstrual volume (P < 0.01), having menarche before 12 years (P < 0.05), and eating breakfast (P < 0.05). In addition, the significant risk factors for PSST functional impairment included PSS (P < 0.001) and menstrual volume (P < 0.01). The significance of the adjusted beta coefficients for general sleep quality (P = 0.054) and drinking (P = 0.054) was close to the significance level.

**Table 5:** Regression coefficients in the multiple regression model including four sleep variables and covariates for dysmenorrhea and premenstrual syndrome

Dependent variables	VAS		CMSS severity		CMSS frequency		No. of PSST symptom		No. of PSST functional impairment	
Independent variables	B (SE)	β	B (SE)	β	B (SE)	β	B (SE)	β	B (SE)	β
Sleep duration	-0.257 (0.370)	-0.031	-1.219 (1.303)	-0.039	-0.456 (1.560)	-0.012	0.641 (0.484)	0.055	-0.056 (0.188)	-0.013
Sleep quality	0.512 (0.269)	0.088	3.274 (0.946)	**0.146** ^**^	4.208 (1.134)	**0.161** ^***^	1.024 (0.351)	**0.125** ^**^	0.264 (0.137)	0.088
Bedtime	0.154 (0.259)	0.026	0.919 (0.912)	0.040	0.659 (1.093)	0.025	0.206 (0.339)	0.025	-0.066 (0.132)	-0.022
Wake-up time	-0.009 (0.262)	-0.002	0.656 (0.923)	0.028	0.444 (1.106)	0.016	0.315 (0.343)	0.037	0.158 (0.133)	0.051
Perceived stress	0.109 (0.021)	**0.233** ^***^	0.508 (0.072)	**0.281** ^***^	0.556 (0.087)	**0.263** ^***^	0.250 (0.027)	**0.376** ^***^	0.067 (0.010)	**0.279** ^***^
Menarche before 12 years	0.434 (0.261)	0.069	1.855 (0.919)	**0.076** ^*^	2.003 (1.100)	0.070	0.744 (0.341)	**0.083** ^*^	0.174 (0.133)	0.053
Family history of dysmenorrhea	0.239 (0.248)	0.040	3.561 (0.872)	**0.155** ^***^	3.618 (1.045)	**0.135** ^**^	0.423 (0.324)	0.050	0.202 (0.126)	0.066
Regularity of menstrual cycle	0.337 (0.252)	0.059	1.930 (0.888)	**0.088** ^*^	0.976 (1.064)	0.038	0.231 (0.330)	0.029	0.189 (0.128)	0.064
Length of menstrual cycle	-0.559 (0.398)	-0.061	-2.373 (1.399)	-0.067	-1.070 (1.676)	-0.026	-0.557 (0.519)	-0.043	-0.270 (0.202)	-0.057
Period length	-0.124 (0.348)	-0.015	-2.040 (1.224)	-0.063	-2.434 (1.466)	-0.065	− 0.041 (0.454)	-0.003	-0.022 (0.177)	-0.005
Menstrual volume	1.369 (0.286)	**0.201** ^***^	5.074 (1.007)	**0.193** ^***^	5.670 (1.207)	**0.185** ^***^	1.051 (0.374)	**0.109** ^**^	0.504 (0.146)	**0.144** ^**^
Eating breakfast	0.257 (0.289)	0.038	3.155 (1.015)	**0.119** ^**^	3.817 (1.216)	**0.124** ^**^	0.837 (0.377)	**0.086** ^*^	0.219 (0.147)	0.062
Smoking	0.157 (0.552)	0.013	2.076 (1.941)	0.043	1.817 (2.325)	0.033	0.667 (0.721)	0.038	0.112 (0.280)	0.018
Drinking	0.183 (0.317)	0.026	2.494 (1.116)	**0.091** ^*^	2.050 (1.336)	0.064	0.571 (0.414)	0.057	0.311 (0.161)	0.085
Caffeine consumption	0.157 (0.243)	0.027	0.583 (0.854)	0.026	1.283 (1.023)	0.049	0.105 (0.317)	0.013	-0.043 (0.123)	-0.014

N = 519. VAS: visual analogue scale, CMSS: Cox menstrual symptom scale, No.: number, PSST: premenstrual symptoms screening tool. Bedtime: 10PM-1AM vs. after 1AM; Wake-up time: 5AM-8AM vs. after 8AM; Sleep duration: < 5 h vs. ≥5 h; Sleep quality: Good vs. poor. ^*^P < 0.05, ^**^P < 0.01, ^***^P < 0.001. The significant values are in bold

## Discussion

The present study explored quantitative and qualitative aspects of sleep on dysmenorrhea and PMS among high school girls aged 15 to 18 years in Korea during the COVID-19 pandemic. The results showed that sleep patterns (i.e., bedtime and wake-up time) was irregular and sleep duration was diverse during the pandemic. Moreover, the prevalence of poor sleep quality was relatively high. Our results from simple regression analyses showed that sleep quality, sleep pattern, and shorter sleep duration had significant effects on dysmenorrhea and PMS. However, after controlling for other risk factors, the effect of bedtime and wake-up time disappeared, while sleep quality still affected dysmenorrhea and PMS and shorter sleep duration affected only PMS. Moreover, our results showed that among components of sleep quality, poor subjective sleep quality, longer sleep latency, frequent sleep disturbance, more daytime dysfunction, and frequent use of sleeping medications worsened dysmenorrhea and PMS. Importantly, to identify the strongest sleep-related predictors of dysmenorrhea and PMS, bedtime, wake-up time, sleep duration, and general sleep quality were simultaneously entered in the multiple regression model with other risk factors. Among sleep variables, sleep quality was the most important risk factor of dysmenorrhea and PMS.

Regarding to sleep characteristics in Korean high school girls during the pandemic, our study showed that about 68% of adolescents slept 7 h or less, about 60% had a poor sleep quality, 64% slept later, and 34% got up later. Similar results were also found in previous studies [51, 52]: Zhou et al. [51] reported that 50.1% of the senior high school students had insufficient sleep (< 7 h) during the COVID-19 pandemic. Da Silva et al. [52] reported that 58.2% of adolescents had worse sleep quality during the pandemic. Additionally, they found that most adolescents had poor sleep quality (68%) and sleep disorders (17.6%), while only 14.4% of adolescents had good sleep quality. Illingworth et al. [36] also reported that in older adolescents, bedtimes and wake times were later in 2020 compared to the 2019 survey, indicating the deterioration of sleep during the national lockdown. The common sleep quality problems included difficulties in falling asleep and maintaining sleep. Interestingly, usage of screen media devices such as smartphones, gaming consoles, and computers in children and adolescents significantly increased during the pandemic, indicating that the increase in media device use during the pandemic could worsen their sleep problems [32]. Indeed, the use of screen media device is related to poor sleep quality and short sleep duration in adolescents [53, 54].

One of the strengths of this study may be that we assessed the multifaceted aspects of dysmenorrhea and quantitative and qualitative characteristics of sleep. Although VAS has been used to assess dysmenorrhea, it is criticized that it may be insufficient for an accurate assessment of dysmenorrhea because VAS measures only pain intensity, whereas dysmenorrhea is multidimensional episode: Various mental and physical symptoms of dysmenorrhea (i.e., nausea, vomiting, diarrhea, headache, backaches, leg aches, dizziness, depression, irritability, nervousness, and so on) often occur [30, 31]. Therefore, dysmenorrhea should be evaluated in a multidimensional manner, including both pain and symptoms [30, 31]. Nevertheless, it was unknown about the effect of sleep on symptoms of dysmenorrhea and premenstrual symptoms in both adolescents and adults.

To examine the effects of sleep pattern and sleep duration on dysmenorrhea, we performed both simple and multiple regression analyses. In our results from simple regression analyses, sleeping late after 1AM worsen pain intensity (VAS) and symptoms of dysmenorrhea (CMSS severity and frequency), while getting up late after 8AM and shorter sleep duration (< 5 h) worsen menstrual symptoms, but not menstrual pain. However, an opposite result was also reported that shorter sleep duration (< 6 h) was a risk factor for the moderate to severe menstrual pain measured by VAS [26]. Thus, further studies may be needed to figure out the difference. When we performed multiple regression to examine the relative importance of sleep pattern and duration compared to other known risk factors of dysmenorrhea, these effects disappeared after controlling for other risk factors of dysmenorrhea, including menstrual and lifestyle factors. Based on our best knowledge, there were no studies on sleep pattern and dysmenorrhea in adolescents and adults.

In our results from simple regression analyses about the effect of sleep quality on dysmenorrhea, general sleep quality (poor vs. good) and 5 PSQI components (i.e., subjective sleep quality, sleep latency, sleep disturbance, daytime dysfunction, and use of sleep medication) significantly affected VAS and the CMSS severity and frequency. When other risk factor entered in the regression model, all these variables of sleep quality still affected the CMSS severity and frequency, while only sleep disturbance affected VAS. However, two PSQI components, sleep duration (score 0–3; >7, 6–7, 5–6, and < 5 h) and sleep efficiency, did not affect dysmenorrhea. Few studies on the effect of sleep quality on dysmenorrhea have been conducted in adolescents: Indeed, only one study reported that adolescent girls with dysmenorrhea had worse insomnia, daytime sleepiness, and sleep apnea [28]. Finally, we conducted multiple regression analyses by entering four sleep variables and other risk factors together as independent variables. The results showed that poor sleep quality, but not late bedtime, late wake-up time, and short sleep duration, was a significant risk factor of CMSS severity and frequency. This implies that among the sleep characteristics, sleep quality was the most important risk factor for symptoms of dysmenorrhea.

The PMS, which start before the period, has been differentiated from dysmenorrhea [55]. Our results of simple regression revealed that sleeping late, getting up late, short sleep duration, poor sleep quality, and 5 PSQI components were significant risk factors of the moderate-to-severe premenstrual symptoms. However, our results of multiple regression analyses showed that after controlling for covariates, short sleep duration, poor sleep quality, and worse 5 PSQI components, but not sleep pattern, increased the number of moderate-to-severe premenstrual symptoms, indicating bedtime and wake-up time were less important risk factors of PMS compared to sleep duration and quality. After controlling for covariates, the number of moderate-to-severe functional impairments due to PMS was increased only by poor sleep quality and worse 5 PSQI components. The other two PSQI components, sleep duration and sleep efficiency, were not significant risk factors of PMS. In part, one similar result was reported, where sleep latency but not sleep efficiency was a risk factor for PMS of Japanese junior high school students aged between 12 and 15 years [29]. Finally, when four sleep variables and other risk factors were entered together in the multiple regression model as independent variables among the sleep characteristics, poor sleep quality was identified as the most important risk factor for PMS symptoms.

It is not well known how sleep affects dysmenorrhea and PMS. Plausible mechanisms may include inflammation, disturbance of reproductive hormones, and stress for their potential relevance to sleep, dysmenorrhea, and PMS [14–17, 56–59]. It has been suggested that in dysmenorrhea, decreased progesterone, overproduced prostaglandin F2α (PGF2α), and increased vasopressin, leukotrienes, and inflammatory factors are considered to result in the excessive contraction and vasoconstriction of the uterus, leading to uterine ischemia and hypoxia and increased the sensitivity of pain fibers [14, 56]. It is plausible that sleep problems can affect menstrual problems by exaggerating inflammation since sleep problems could increase inflammation, which is one of the pathophysiological causes of dysmenorrhea. In a previous meta-analysis study, it has been reported that sleep disturbance and long sleep duration, but not short sleep duration, are associated with increases in markers of systemic inflammation [15]. Additionally, melatonin is also known to be associated with reproductive health [18, 19]: It has been reported that sleep disturbance decreases melatonin level [20] and abnormal secretion of melatonin was found in women with PMS [20]. Meanwhile, the possibility that stress partly mediates the effect of sleep on dysmenorrhea and PMS cannot be ruled out because poor sleep aggravates stress, a risk factor of dysmenorrhea and PMS [8, 16, 17]. However, our results suggested that sleep may independently affect dysmenorrhea and premenstrual symptoms because sleep quality had a significant effect after controlling for the effect of perceived stress.

While there appears to be an association between poor sleep and dysmenorrhea/PMS in adults, it is less known about this in adolescents. Furthermore, little is known about which aspects of sleep are related to dysmenorrhea and PMS and which aspects of dysmenorrhea and PMS are particularly affected in both adults and adolescents. In the present study, we showed that in adolescents, poor sleep quality was the most important risk factor of dysmenorrhea and PMS and along with pain, menstrual symptoms were also affected by poor sleep quality.

However, our study had several limitations as follows: Firstly, there was a possibility of selection bias because the convenience sampling method was used. Specifically, those who had dysmenorrhea were more likely to agree to participate than those who did not have such problems. Secondly, because this was a cross sectional study, we were unable to establish causal inferences. That is, it is unclear whether menstrual problems result in sleep problems, or the other way around. Future studies using a longitudinal design or case-control design are thus needed to provide causal relationship evidence. This may restrict the generalizability of the present findings.

## Conclusions

This study highlights the importance of healthy sleep hygiene, especially good sleep quality in adolescents, to manage dysmenorrhea and PMS. Being conducted during the COVID-19 pandemic, this study’s findings may be limited in their generalizability. Nonetheless, to the best of our knowledge, our research is the first study that provides comprehensive insight into the impact of sleep characteristics on the menstrual health of adolescents. Through this, the evaluation of sleep characteristics should be considered in adolescents when prescribing or selecting interventions to alleviate dysmenorrhea and PMS. In addition, this study aims to raise awareness of the importance of good sleep quality for adolescents to relieve dysmenorrhea and PMS.

## Data Availability

The data are available from the corresponding author on a reasonable request.

## List of abbreviations

CMSS: Cox menstrual symptom scale
COVID-19: Coronavirus disease 2019
PMS: Premenstrual syndrome
PSST: Premenstrual symptoms screening tool
PSQI: Pittsburgh Sleep Quality Index
PSS: Perceived stress scale
VAS: Visual analogue scale
